# Microbiome Variation Across Populations of Desert Halophyte *Zygophyllum qatarensis*

**DOI:** 10.3389/fpls.2022.841217

**Published:** 2022-03-31

**Authors:** Abdul Latif Khan, Lucas Dantas Lopes, Saqib Bilal, Sajjad Asaf, Kerri M. Crawford, Venkatesh Balan, Ahmed Al-Rawahi, Ahmed Al-Harrasi, Daniel P. Schachtman

**Affiliations:** ^1^Department of Engineering Technology, College of Technology, University of Houston, Sugar Land, TX, United States; ^2^Natural and Medical Sciences Research Centre, University of Nizwa, Nizwa, Oman; ^3^Department of Agronomy and Horticulture, Centre for Plant Science Innovation, University of Nebraska-Lincoln, Lincoln, NE, United States; ^4^Department of Biology and Biochemistry, College of Natural Science and Mathematics, University of Houston, Houston, TX, United States

**Keywords:** microbiome, desert succulents, *Zygophyllum qatarensis*, microbial communities, microbial diversity, core-microbiome

## Abstract

Microbial symbionts play a significant role in plant health and stress tolerance. However, few studies exist that address rare species of core-microbiome function during abiotic stress. In the current study, we compared the microbiome composition of succulent dwarf shrub halophyte *Zygophyllum qatarensis* Hadidi across desert populations. The results showed that rhizospheric and endosphere microbiome greatly varied due to soil texture (sandy and gravel). No specific bacterial amplicon sequence variants were observed in the core-microbiome of bulk soil and rhizosphere, however, bacterial genus *Alcaligenes* and fungal genus *Acidea* were abundantly distributed across root and shoot endospheres. We also analyzed major nutrients such as silicon (Si), magnesium, and calcium across different soil textures and *Z. qatarensis* populations. The results showed that the rhizosphere and root parts had significantly higher Si content than the bulk soil and shoot parts. The microbiome variation can be attributed to markedly higher Si – suggesting that selective microbes are contributing to the translocation of soluble Si to root. In conclusion, low core-microbiome species abundance might be due to the harsh growing conditions in the desert – making *Z. qatarensis* highly selective to associate with microbial communities. Utilizing rare microbial players from plant microbiomes may be vital for increasing crop stress tolerance and productivity during stresses.

## Introduction

Arid land ecosystems cover over 30% of earth and are inhabited by nearly a billion people. Both plant and microbial life are confronted with extreme living conditions that greatly depend on a scarcity of water and nutrients from soil. In plants, xerophytic succulents and annuals are the key species well-tailored to continuous episodes of abiotic stresses (drought, heat, and salinity) (Ndour et al., 2020; Peguero-Pina et al., 2020; Zeng et al., 2021). Succulent plants are common in arid land ecosystems due to their ability to store significant amounts of water in cells, their potential to withstand or avoid extreme drought periods, and growth patterns (Griffiths and Males, 2017; Heyduk, 2021). In addition, the survival of succulent plants in arid environments is often attributed to their (i) genetic makeup, (ii) physio-photosynthetic responses, (iii) essential metabolite production, and (iv) associated microbial symbionts (Heyduk, 2021). In the latter case, the mutualistic microbiota (mainly bacteria and fungi) inhabiting the soil around roots (rhizosphere) and the shoot surface (phyllosphere) play a considerable role in improving physiological responses, beneficial metabolite production, and nutrient uptake (Khan et al., 2015, 2020; Trivedi et al., 2020). Though the phytobiome consists of a great diversity of micro and macroorganisms in and around plants, disentangling the effects of the phytomicrobiome on plant performance has emerged as a potential solution for economically important plants to deal with changing global climate and improve productivity and disease resistance (D’Hondt et al., 2021).

The plant-associated microbiome has been coined as the plant’s “second genome” that is highly variable in diversity, abundance, and composition (Berendsen et al., 2012). This microbial variability is due to (i) abiotic factors like temperature, water (wet or dry), soil chemistry, and nutrient cycling, (ii) the plant species, developmental stage, its ability to establish successful associations with the microbiome, the interaction of the microbiome with hub microbiota and keystone species, and (iv) how root exudates influence microbial growth and reproduction (Trivedi et al., 2020; D’Hondt et al., 2021). There has been a considerable effort to elucidate the plant-microbiome interactions and the microbial niches in arid land ecosystems. Indeed, extreme environments may contain highly beneficial culturable microbes that can, in turn, withstand the adverse impacts of stressful conditions. However, interactions between the microbial communities and succulent plants have been minimally investigated, particularly in arid ecosystems (Pfeiffer et al., 2017). Previous studies (Jorquera et al., 2016; Citlali et al., 2018; Delgado-Baquerizo et al., 2018; Mandakovic et al., 2018; Araya et al., 2020; Astorga-Eló et al., 2020; Khan et al., 2020) have evaluated the microbiome, especially bacterial communities from arid soil. However, little work has explored the phyllosphere and rhizosphere microbiome across different populations of the same host plant species. Notably, more diversity in sampling may provide additional significant insights into the plant microbiome and help discover new beneficial microbes.

The Earth Microbiome Project has estimated nearly 10 million microbial species globally, whereas other estimates suggest a trillion species (Locey and Lennon, 2016; Thompson et al., 2017). However, only a small fraction of the Earth microbial species has been sequenced or are available in culture stocks. Hence, there is a great need to explore the unique phytomicrobiome and the keystone species of extreme arid environments. Some of the succulents recently analyzed for their microbiome are *Agave* species (Flores-Núñez et al., 2020), *Aloe vera* (Akinsanya et al., 2015), cacti (Fonseca-García et al., 2016), CAM plants (Citlali et al., 2018), pineapple (Putrie et al., 2020), and Aizoaceae (Pieterse et al., 2018). These microbiome studies showed remarkably high and diverse rhizosphere colonization by the bacterial phyla *Actinobacteria, Proteobacteria, Firmicutes, Actinobacteria, Acidobacteria*, and *Bacteroidetes* (Citlali et al., 2018; Flores-Núñez et al., 2020). However, the importance of understanding the microbiome composition of wild plants growing in arid environments has been underappreciated until recently.

In the present study, we investigated the phytomicrobiome of salt-tolerant dwarf shrub *Zygophyllum qatarensis* Hadidi (Basionym of *Z. hamiense* var *qatarensis*, *Tetraena qatarensis*) and its four major populations. *Z. qatarensis* is a drought and salinity resistant plant endemic to the Arabian Peninsula (Beier et al., 2003; Alzahrani and Albokhari, 2017), where it grows on coarse, stony, or calcareous sandy soils (Sayed, 1996; Abbas, 2005). The plant grows well in arid desert ecosystems despite exposure to high drought, heat, and intense UV conditions. The leaves are fleshy and succulent and can store enough water to sustain plants through arid periods. However, the unifoliate xeromorphic leaf morphology changes depending on water availability (Sayed, 1996; Abbas, 2005). The leaves gradually shed depending upon the intensity of drought and heat in desert conditions. The seed has a tough outer coat and only germinates upon a considerable amount of rainfall. The immature leaves are used by humans as a vegetable and possess several medicinal properties used to treat diabetes and dysmenorrhea. The plant sources biologically active phytochemicals such as terpenoids, phenolics, and essential oils (Zaman and Padmesh, 2009; Shawky et al., 2019). In addition, due to their outstanding resistance to arid conditions, the unique *Z. qatarensis* microbiome has a high potential for identifying beneficial microbial strains that aid crop drought-stress tolerance. Herein, we investigated for the first time the rhizosphere and root/shoot endosphere microbiome of *Z. qatarensis* to describe its microbiome across different populations growing in two different soil conditions (gravel and sandy).

## Materials and Methods

### Soil, Rhizosphere, and Plant Endosphere Sampling

**FIGURE 1:**
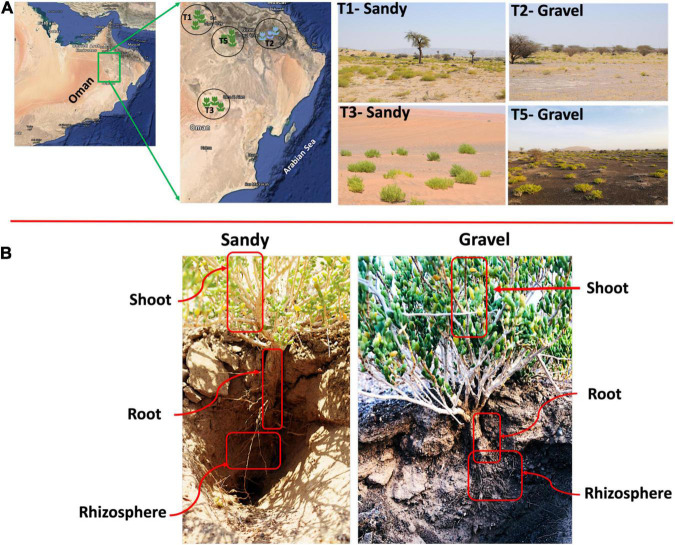
Habitat of *Z. qatarensis*. **(A)** Location map of samples across different parts of desert ecosystem where *Z. qatarensis* grows. Mainly two populations (growing in gravel and sandy soils) were scattered from north to southern regions. **(B)** Samples were collected from bulk soil, rhizosphere, and root/shoot endosphere of sandy and gravel populations.

The *Z. qatarensis* plants (shoot and root), rhizosphere and bulk soil were collected from four major population locations (T1, T2, T3, and T5) in the extreme desert of Empty Quarter in Oman (Supplementary Table 1 and Figure 1). The *Z. qatarensis* plant populations are distributed in a conserved and consistent ecological pattern across the four distinctive locations in desert areas. Each population was approximately 100 km apart and samples within a population were collected in triplicate. Each replica from the individual population was representative of ten plants/soils that were pooled for DNA extractions and chemical analysis. The rhizosphere soils adjacent to the root surface (10 to 15cm deep) were collected (Supplementary Figure 1). These were mature plants with a reasonably defined rooting system and the sandy soils were removed with the help of a sterile blade. There were no specific signs of rhizosheath with the root, so the soil attached to root parts was categorized as rhizosphere – following the classification of Pang et al. (2021). The bulk soil samples were collected from a depth of 10 to 15cm with no signs of *Z. qatarensis* presence. The root parts were carefully collected by removing the sand and particulate matters. For endosphere, the root and shoot samples were washed with sterile distilled water and sodium hypochlorite to remove epiphytic microbes following the method of McPherson et al. (2018). Briefly, the pre-sterilized scalpel (with 70% ethanol) was used to prepare individual tissues from about 10 to 15 roots and shoot parts ranging from 4 to 6 cm in length. The excised tissues were placed in autoclaved phosphate buffer (NaH_2_PO_4_ − 6.33 g/L, Na_2_HPO_4_ − 8.5 g/L with pH = 6.5: McPherson et al. (2018). The sterilized samples were stored at − 20°C for DNA extractions. All the samples were stored at 4°C for soil chemical analysis. The sampling area’s climate is dry with annual rainfall lower than 30 mm and summer temperature can reach up to +48°C with a relative humidity of 20–30% (Supplementary Table 2). The samples were collected during the dry summer season (May–June 2020).

### DNA Extraction, Library Preparation, and MiSeq Sequencing

After pooling the soil/plant samples within each replicate, 10.0 g mixtures were subjected to total DNA extraction using the MoBio Power Soil DNA Extraction Kit. The DNA was quantified using Qubit 4.0 and high sensitivity kit (Invitrogen, United States). Libraries of each DNA sample were generated by amplifying the internal transcribed spacer (ITS region) and 16S rRNA gene (V4 region) using Nextflex PCR I Primer Mix (Perkin Elmer, United States) for fungal and bacterial communities, respectively (Supplementary Figure 2). Illumina amplicon primers for 16S (Forward 5′–TCGTCGGCAGCGTCAGATGTGTATAAGAGACAGCCTACG GGNGGCWGCAG–3′; Reverse 5′– GTCTCGTGGGCTCG GAGATGTGTATAAGAGACAGGACTACHVGGGTATC TAA TCC –3′) and ITS (Forward 5′–AATGATACGGCGACCAC CGAGATCTACACGG CTTGGTCATTTAGAGGAAGTAA–3′; Reverse 5′–CAAGCAGAAGACGGCATACGAGA TCGGCTG CGTTCTTCATCGATGC–3′) were used. For the 16S rRNA gene, peptide nucleic acid (PNA) clamps were used to reduce mitochondrial and chloroplast contamination.

A paired-end sequencing approach with read lengths of 250 bp was conducted on an Illumina MiSeq instrument (Illumina Inc., San Diego, CA, United States) operating with v2 chemistry (User Guide Part # 15027617 Rev. L). All quality reads related to the study are available at NCBI under BioProject PRJNA771947 (SRP341951) and PRJNA767523 (SRP339516) for bacteria and fungi, respectively.

### Bioinformatics Analysis of Sequencing Reads

The sequencing reads were analyzed with QIIME 2.0 (Bolyen et al., 2019). First, reads from ITS and 16S rRNA amplicons were separated into different files. Then, the average quality of forward and reverse reads was observed in each dataset. Only the forward reads were used for the following analyses due to the low quality of the reverse reads in both datasets. We used the DADA2 algorithm for denoising and generating the amplicon sequence variants (ASV) table for each dataset (Callahan et al., 2016). The reads clustered in the same ASV have nucleotide sequences that are 100% identical (Callahan et al., 2017). In the denoising, sequences were filtered by overall quality, trimmed in low-quality regions, and chimeric sequences were removed. The 16S rRNA gene reads were trained on the SILVA database for taxonomic classification (Quast et al., 2012), while the UNITE database was used to classify the ITS sequences (Nilsson et al., 2019). Sequences classified as mitochondria and chloroplast were removed from the 16S rRNA gene ASV table. The 16S rRNA gene and ITS ASV tables were rarefied to 6000 and 100 reads for diversity analyses containing all samples from each dataset, respectively. The ASV tables of each dataset were then split for each sampling compartment for a more detailed analysis. For the bacterial diversity analyses, the bulk soil, rhizosphere, root endosphere, and shoot endosphere ASV tables were rarefied to 30,000, 37,000, 7,500, and 6,200 reads, respectively. For the fungal diversity analyses, the bulk soil, rhizosphere, root endosphere, and shoot endosphere ASV tables were rarefied to 120, 100, 1,300, and 1,200 reads, respectively. For beta-diversity analyses, the Bray-Curtis distance matrix was generated for each dataset and exported to statistical software. For alpha-diversity analyses, the Shannon index and Observed ASVs were calculated in each dataset to infer species diversity and richness, respectively.

### Microbiome Analyses

The Bray-Curtis distance matrices were exported to R for multivariate statistical analyses. Non-metric multidimensional scaling (NMDS) was performed with the metaMDS function to show the ordination of samples according to the major factors (plant compartment) affecting the microbial communities, while constrained analysis of principal coordinates (CAP) was performed with the capscale function to show the ordination of samples according to more specific factors (sampling locations and soil type) using the “vegan” package v. 2.5-6 (Dixon, 2003). Both NMDS and CAP results were visualized with the ggplot function in the “ggplot” package v. 3.3.0 (Wickham, 2011). Permutative multivariate analysis of variance (PERMANOVA, 999 permutations) was used to test for significant effects of the factors studied (plant compartment, sampling location and soil type) and their interaction on bacterial and fungal beta-diversity using the “adonis function.” Analysis of similarity (ANOSIM) was also used as a second method to test the effects of the factors studied on the fungal and bacterial communities using in R. Differences in species diversity (Shannon index) and richness (Observed ASVs) for the same factors were assessed using the Kruskal-Wallis test in QIIME 2.0 (Bolyen et al., 2019). Changes in the relative abundance of microbial genera between soil types were evaluated with the Kruskal-Wallis test and Bonferroni *p*-value correction using STAMP software (Parks et al., 2014).

### Chemical Properties and Nutrient Composition

The four populations were sampled from soils characterized as sandy (T1/T3) and gravel (T2/T5), where the pH ranges from 6.7 to 7.2 and electrical conductivity from 27 to 39 dS m^–1^. The soils with more gravel structure have higher nitrates than sandy ones (Supplementary Table 2). A detailed soil chemical analysis was performed (Khan et al., 2020). Furthermore, essential nutrients such as silicon (Si), magnesium (Mg) and calcium (Ca) were quantified from plants and soil from the four populations as described previously (Bilal et al., 2018) using inductively coupled plasma mass spectrometry (ICP-MS; Optima 7900DV, Perkin-Elmer, United States). All the measurements were carried out in triplicate.

### Statistical Analysis

At least three replicate samples were analyzed during this study. The data for the enzyme study is presented as the mean ± standard error (SEM). The significant differences were determined using one-way analysis of variance (ANOVA). The differences were considered significant at *P* < 0.05 and were calculated by GraphPad Prism Version 6.01 (GraphPad Software, San Diego, CA, United States). Duncan’s multiple range test at *P* < 0.05 (SAS 9.1, Cary, NC, United States) was used to compare the mean values.

## Results

### Shifts in Microbial Community Structure and Diversity Between Plant Compartments, Sampling Locations, and Soil Types

**FIGURE 2:**
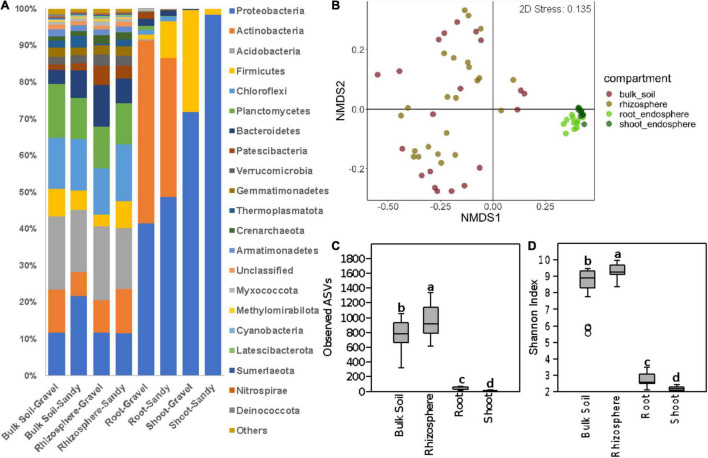
Bacterial community composition and diversity in all sampling compartments. **(A)** Bar charts show the relative abundance of the dominant phyla in bulk soil, rhizosphere, root endosphere, and shoot endosphere samples of plants growing in gravel or sandy soils. “Others” stand for the sum of other phyla with <1.5% of relative abundance. **(B)** Bacterial community structure represented by a non-metric multidimensional scaling (NMDS) ordination based on the Bray-Curtis distance matrix of the 16S rRNA gene ASV table. Samples are colored according to the sampling compartment. **(C,D)** Differences in bacterial alpha-diversity between the four plant and soil compartments. **(C)** Bacterial species richness based on the number of observed ASVs. **(D)** Bacterial species diversity based on the Shannon index. Different letters indicate significant differences according to the Kruskal-Walli’s test (*p* < 0.05).

A total of 58,648 ITS and 2,715,959 16S rRNA sequence reads passed all quality filters and were used for the following analyses. We analyzed the differences in bacterial/archaeal and fungal community structure between four locations where *Z. qatarensis* was naturally found (Figure 1A). Location T1 and T3 have sandy soils, while locations T2 and T5 have gravel soils (Figure 1A). The microbiomes of two soil (bulk soil and rhizosphere) and two plant endosphere (roots and shoots) compartments were investigated (Figure 1B and Supplementary Figure 3). The bacterial/archaeal community was dominated by the phylum Proteobacteria in the root and shoot endosphere compartments and Acidobacteria in the rhizosphere and bulk soil compartments (Figure 2A). Proteobacteria had a higher relative abundance in sandy than gravel soils in the bulk soil and shoot endosphere. In the rhizosphere and root endosphere compartments, the phylum Firmicutes was relatively more abundant in sandy than in gravel soils (Figure 2A). On the other hand, in the shoot endosphere *Firmicutes* were proportionally more abundant in gravel than in sandy soils (Figure 2A). PERMANOVA results indicated that sampling compartment was the main factor significantly affecting (*p* < 0.001; *R*^2^ = 0.34) the bacterial/archaeal communities, followed by location (*p* < 0.001; *R*^2^ = 0.08). The interaction effect between compartment and location was also significant (*p* = 0.031; *R*^2^ = 0.12) on the bacterial communities. The bulk soil and rhizosphere samples did not show a clear separation in the NMDS, but clustered separately from the root and shoot endosphere samples (Figure 2B). ANOSIM results also showed a significant effect of compartments (*p* < 0.001; *R* = 0.53) and locations (*p* = 0.022; *R* = 0.34) on the bacterial communities. When analyzing the samples from all compartments together, soil type was not a significant factor in shaping the bacterial community structure according to PERMANOVA and ANOSIM.

**FIGURE 3:**
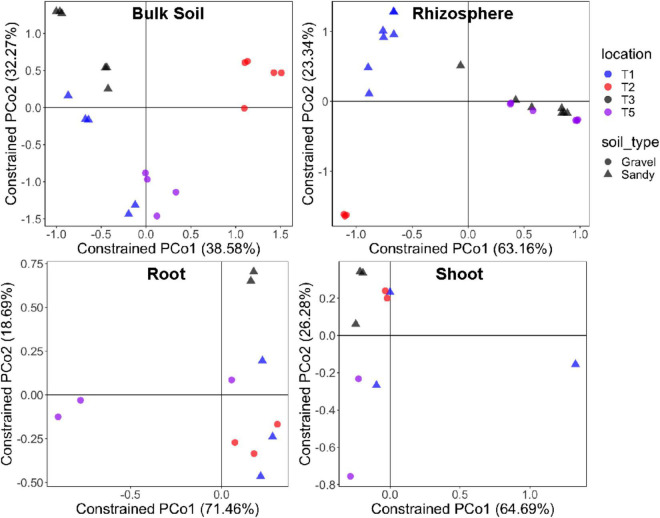
Bacterial community structure in each specific sampling compartment. Constrained analysis of principal coordinates (CAP) based on the Bray-Curtis distance matrix of the 16S rRNA gene ASV table, showing the ordination of samples according to the factors sampling location and soil type in each compartment separately. The percentage of variation explained by the two first axes is provided for each graph. Different colors stand for the four locations, while different symbols stand for the two soil types.

Bacterial alpha-diversity was also significantly different between compartments, with greater species richness and diversity in the rhizosphere, followed by bulk soil, root endosphere, and shoot endosphere (Figures 2C,D). The bacterial community distribution and abundances across different soil types and samples have also been shown through heat and networking maps (Supplementary Figures 4, 5). The analysis of each compartment separately indicated that the bacterial community structure was significantly different between the gravel and sandy soils in the rhizosphere (*p* = 0.025; *R*^2^ = 0.07) and bulk soil (*p* = 0.013; *R*^2^ = 0.09), but locations had a larger effect than soil type for both rhizosphere (*p* < 0.001; *R*^2^ = 0.29) and bulk soil (*p* = 0.02; *R*^2^ = 0.17) (Figure 3). Location T2 had the most different bacterial community composition among the four locations (Figure 2). The root and shoot endosphere bacterial community structure was not affected by soil type and location. The alpha diversity was not affected by soil type and locations in any of the four compartments.

**FIGURE 4:**
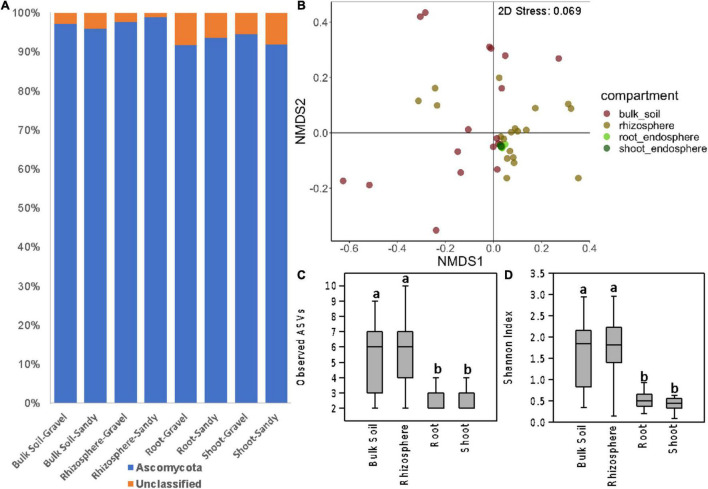
Fungal community composition and diversity in all sampling compartments. **(A)** Bar charts showing the relative abundance of fungal phyla in bulk soil, rhizosphere, root endosphere, and shoot endosphere samples of plants growing in gravel or sandy soils. **(B)** Fungal community structure represented by a non-metric multidimensional scaling (NMDS) ordination based on the Bray-Curtis distance matrix of the ITS ASV table. Samples are colored according to the sampling compartment. **(C,D)** Differences in fungal alpha-diversity between the four plant and soil compartments. **(C)** Fungal species richness based on the number of observed ASVs. **(D)** Fungal species diversity based on the Shannon index. Distinct letters indicate significant differences according to the Kruskal-Walli’s test (*p* < 0.05).

The Ascomycota phylum dominated the fungal community in all samples from the four compartments (Figure 4A). This dominance was mainly represented by the ascomycete species *Acidea extrema*. The root and shoot endosphere compartments had proportionally more unclassified sequences than the bulk soil and rhizosphere (Figure 4A and Supplementary Figure 6). As observed for the bacterial community, the fungal community structure was more affected by plant compartment (*p* < 0.001; *R*^2^ = 0.21), followed by sampling location (*p* = 0.014; *R*^2^ = 0.08) according to PERMANOVA. The interaction between these two factors also significantly affected the fungal communities (*p* = 0.032; *R*^2^ = 0.16). However, ANOSIM indicated that only location (*p* = 0.028; *R* = 0.05), but not compartment (*p* = 0.119; *R* = 0.05) significantly affected the fungal community structure. The separation between samples from the four compartments was unclear in the NMDS ordination (Figure 4B). Soil type did not affect the fungal beta-diversity when analyzing the samples from the four compartments together.

**FIGURE 5:**
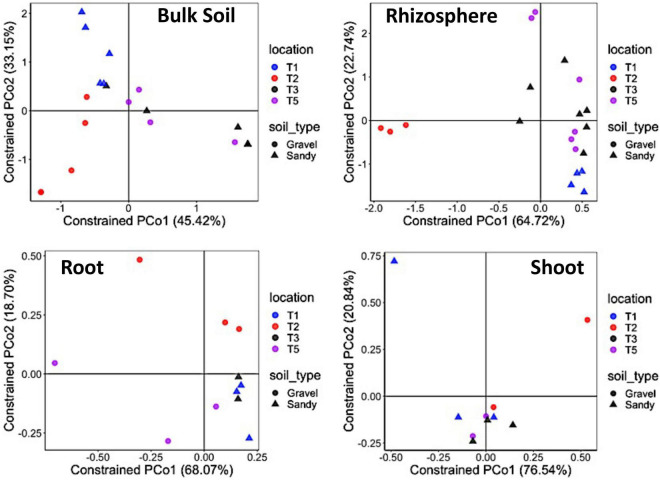
Fungal community structure in each specific sampling compartment. Constrained analysis of principal coordinates (CAP) based on the Bray-Curtis distance matrix of the ITS ASV table shows samples’ ordination according to the sampling location and soil type in each compartment separately. The percentage of variation explained by the two first axes is provided for each graph. Different colors stand for the four locations, while different symbols stand for the two soil types.

As seen for bacteria, the fungal alpha-diversity was also affected by plant compartments, with greater species richness and diversity in the rhizosphere and bulk soil than in the root and shoot endosphere (Figures 4C,D). It is noteworthy the low fungal alpha diversity in all compartments. Rarefaction curves showed that the sequencing depth and sample rarefaction (100 reads) used were sufficient to reflect the fungal diversity in these samples (Supplementary Figure 7). In addition, the species richness was also affected by different locations, with greater values in T3 and T5 than in T1 and T2 (Supplementary Figure 5). When separating the analysis by each compartment, soil type still did not affect the fungal beta-diversity in any compartment. Soil type also did not affect the fungal alpha-diversity in any compartment. However, location significantly affected the fungal community structure of the rhizosphere (*p* < 0.001; *R*^2^ = 0.37) and bulk soil (*p* = 0.049; *R*^2^ = 0.23) compartments (Figure 5). Like what was observed for bacteria, the fungal communities of location T2 were the most different among the four locations, mainly in the rhizosphere (Figure 5). Moreover, the fungal species diversity decreased in T2 compared to T3 and T5 in the rhizosphere compartment (Supplementary Figure 5).

### The Core Microbiome of *Zygophyllum qatarensis* and Differences in the Relative Abundance of Specific Microbial Genera Between Soil Types

After analyzing the microbiome differences between locations and soil types, we aimed to identify the core microbiome of *Z. qatarensis* regardless of soil type and geographical location. We considered the core microbiome to be the microbes shared by >90% of all plant samples along with the four locations. Results indicated that no specific bacterial ASV was in the core microbiome of the bulk soil, rhizosphere, and root endosphere compartments, but one ASV classified in the *Alcaligenes* genus was present in the shoot endosphere of all plant samples. Similarly, no fungal ASV was present in the bulk soil and rhizosphere core microbiomes. However, two ASVs were found in the root endosphere core microbiome and three ASVs in the shoot endosphere core microbiome. One of these ASVs was in the core microbiome of both root and shoot endosphere and was classified in the species *Acidea extrema*, while the others were from unknown fungal genera. Next, we analyzed the core microbiome at the genus level. A total of 27 bacterial genera were in the bulk soil core microbiome, including *Bryobacter*, *Chthoniobacter*, *Gemmata*, *Nitrospira*, *Pirellula*, *Rubrobacter* and other 21 unknown/undescribed genera. On the other hand, no bacterial genus was in the rhizosphere core microbiome, while only one genus was in the root and shoot endosphere core microbiomes: *Alcaligenes*. Only the fungal genus *Acidea* (species *A. extrema*) was in the core microbiomes of all compartments. These results indicate that the different locations and soil types significantly affect the microbial beta-diversity and the core microbiome – shrinking it to none or just a few microbes.

**FIGURE 6:**
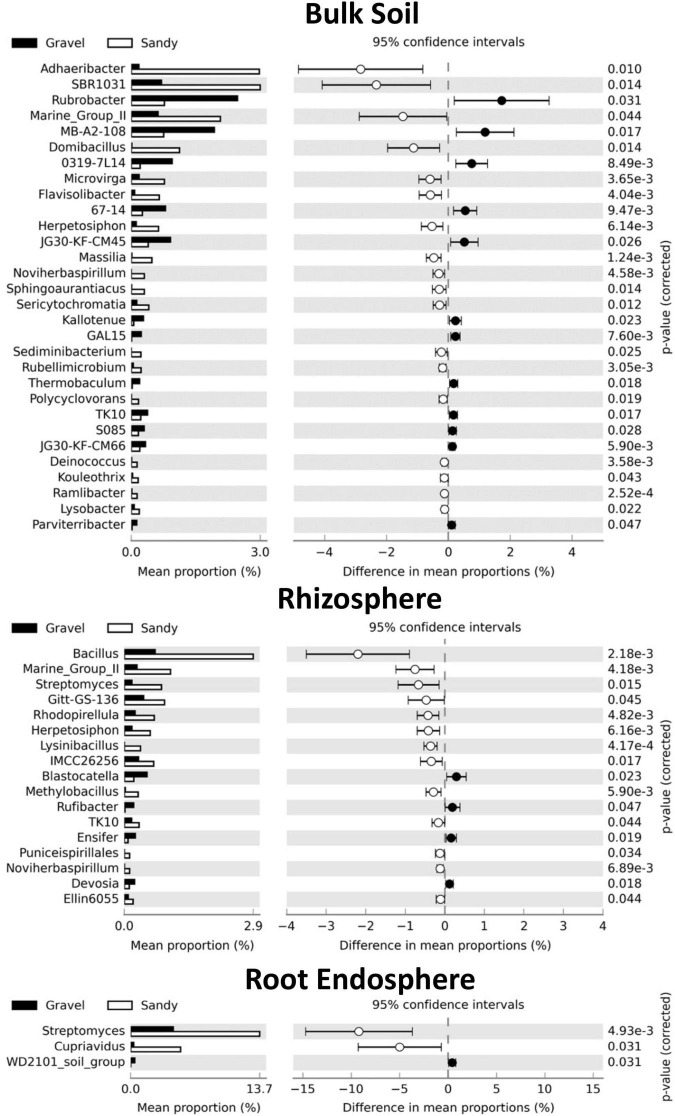
Differences in the relative abundance of specific bacterial genera between the two soil types in each compartment. Set of bacterial genera that significantly shifted in relative abundance according to the Kruskal-Wallis’s test with the Bonferroni *p*-value correction (*p* < 0.05) in each sampling compartment. The genera are sorted based on the largest differences in the mean proportion of sequences between samples collected from gravel (black) and sandy (white) soils. The shoot endosphere was not shown in the figure because no significant differences were found in this compartment.

We further investigated the changes in the microbial communities between the soil types by analyzing shifts in the relative abundance of specific bacterial and fungal genera. The bulk soil compartment had the highest number of bacterial genera (30) with significantly different relative abundances between the gravel and sandy soils, from which 18 were enriched in the sandy soils and 12 were enriched in the gravel soils (Figure 6). The bacterial genus with the largest relative abundance in sandy soils compared to gravel soils was *Adhaeribacter*. The genus with the largest relative abundance in gravel compared to sandy soils was *Rubrobacter* (Figure 6). The rhizosphere was the second compartment with more changes in the relative abundance of specific bacterial genera (17) between the soil types, where 13 genera were enriched in the sandy soils and four genera were enriched in the gravel soils (Figure 6). The bacterial genus with the most extensive proportional enrichment in the sandy soils was *Bacillus*. *Blastocatella* was the bacterial genus with the most significant relative abundance in gravel soils compared to sandy soils (Figure 6). The root endosphere showed only three changes in the relative abundance of bacterial genera between the soil types, including the enrichment of *Streptomyces* and *Cupriavidus* in the sandy soils and the putative genus WD101_soil_group in the gravel soils (Figure 6).

The genera affected by soil types were generally not the same in the rhizosphere and bulk soil, except *Noviherbaspirillum, Marine_Group_II, and Herpetosiphon*, which were enriched in the sandy soils in both compartments (Figure 6). Furthermore, *Streptomyces* was enriched in sandy soils in both rhizosphere and root endosphere (Figure 6). No bacterial genera had different relative abundances between soil types in the shoot endosphere compartment. No fungal genera changed in relative abundance between soil types in any of the four compartments. Many bacterial genera also had differences in relative abundance between locations within each soil type, but there were more changes in the rhizosphere than in the bulk soil (Supplementary Figures 4, 5). The root and shoot endosphere did not show any differences in genera between locations, and no fungal genera differences in relative abundance between locations in any compartment.

### Nutrient Assimilation in Rhizosphere and Phyllosphere of *Zygophyllum qatarensis*

**FIGURE 7:**
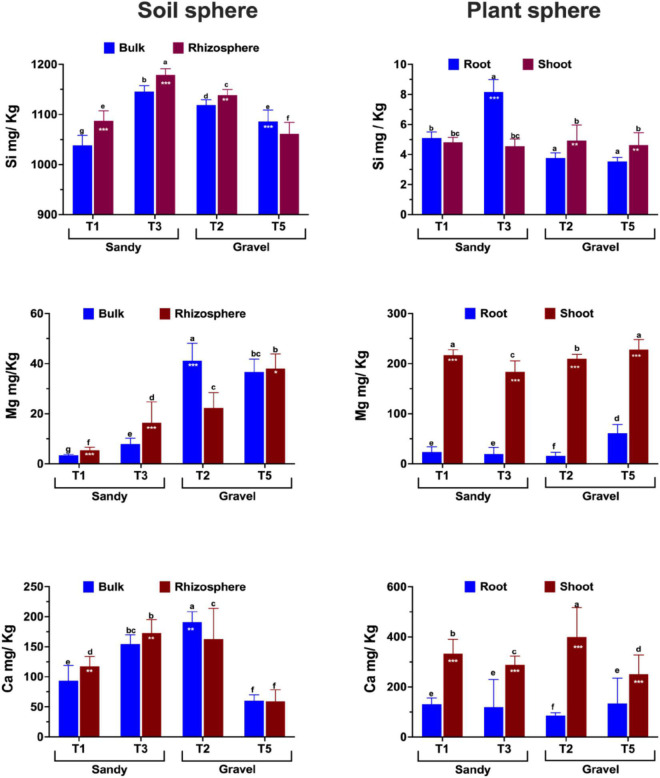
Essential plant growth-related nutrient composition and abundance across soil (bulk and rhizosphere) and plant parts (shoots and roots) of different populations of *Z. qatarensis*. The error bars represent standard deviations. The different letter(s) on the graph indicate significant differences between treatments (*p* < 0.05), as revealed by the DMRT test on SAS 9.0. The *p < 0.05, **p < 0.001, and ***p < 0.0001 on selected bars indicate significant effects of drought treatment.

Three major nutrients, *i.e.*, silicon (Si), magnesium (Mg), and calcium (Ca), were quantified in the soil, rhizosphere, and shoots across four populations of *Z. qatarensis*. In addition to major nutrients, we also assessed the soil physical and morphological properties shown in Supplementary Figure 8. The results showed that the rhizosphere had significantly (*p* < 0.01) higher Si content than bulk soil. Among the populations growing in each soil type, T3 had a significantly higher Si content than T1 in sandy soils, whereas T2 had a significantly higher Si content than T5 in gravel soils (Figure 7). The rhizosphere of T1, T3, and T2 had 23, 21.5, and 15.8% more Si than bulk soil. However, the rhizosphere of T5 had a significantly lower Si content compared to bulk soil. In the case of root to shoot parts, T1 and T3 (sandy soils) had significantly higher (*p* < 0.05; 8.7 and 41.6%, respectively) Si content in roots compared to T2 and T5 (gravel soils). On the other hand, the T2 and T5 had significantly higher (*p* < 0.05; 13.2 and 11.8%, respectively) Si in shoots compared to roots (Figure 7).

In the case of Mg, T2 (gravel texture) had a significantly higher (*p* < 0.003; 44.7%) content in bulk soil, while T5 had a higher content in rhizosphere. In contrast, T1 and T3 (sandy) had significantly higher (*p* < 0.001; 4.2 and 21.8% respectively) Mg content in the rhizosphere than in bulk soil. Overall, Mg amounts were more elevated in gravel than in sandy soils across the different populations. In plants, the Mg content was 7- to 8-fold higher (*p* < 0.001) in shoots than in roots. Among populations, shoots of T1 and T5 had a significantly higher Mg content than T3 and T2. Only T5 had a higher Mg content than T1, T2, and T3 (Figure 7).

In the case of Ca, it was significantly higher in the rhizosphere of T1 (8.7%) and T3 (7.2%) compared to the sandy bulk soils (T2 and T5) of *Z. qatarensis*. Conversely, in gravel soil, the T2 population of *Z. qatarensis* had a significantly higher (14.2%) Ca content in bulk soil compared to rhizosphere. T5 had lower Ca than the other populations of *Z. qatarensis* growing in different locations. In the case of the plants’ organs, shoots of *Z. qatarensis* had in general, a significantly higher Ca content than roots. A considerably higher amount of Ca was observed in T2 > T1 > T3 > T5 in shoots. This increase was four to fivefold higher than in roots (Figure 7).

## Discussion

There has been an unprecedented emphasis on understanding and exploring unique microbiomes and elucidating their function for greater human benefits in agriculture. This study explored the microbiome associated with the desert halophyte *Z. qatarensis* in four populations growing in two soil types in four different locations. We hypothesized that either location or soil type would influence the microbial community structure. Furthermore, we generated the first endosphere datasets for this species, which could help us understand whether and how the plant’s microbiome increases its survival in the harsh desert environment. The results clearly showed that the microbial communities’ distribution and diversity were significantly affected by location and soil types (sandy and gravel). This is consistent with previous studies showing that the rhizosphere and root endosphere microbial communities are affected by environmental factors such as soil pH, salinity, moisture content, soil leaching, erosion, and loss of certain nutrients (Mukhtar et al., 2018, 2021; Khan et al., 2020; Bossolani et al., 2021) since different locations and soil types usually show changes in soil physico-chemical factors.

There are several studies on the microbiomes of extreme desert environments such as those found in the Atacama Desert (Araya et al., 2020; Contador et al., 2020; Menéndez-Serra et al., 2020), Lejía Lake (Mandakovic et al., 2018), the Empty Quarters in Oman (Khan et al., 2020), the Sonoran Desert (Andrew et al., 2012; Finkel et al., 2012; Gornish et al., 2020), the Mojave Desert (Pombubpa et al., 2020), the Saline Lakes of Monegros Desert, Spain (Menéndez-Serra et al., 2020), Eastern Mediterranean (Mazar et al., 2016), and the seed-associated microbiome from Southern Chihuahua Desert (Menéndez-Serra et al., 2020). Some succulent and arid-land plant species were also recently explored for their microbiome structures such as *Agave* spp. (Flores-Núñez et al., 2020), *Aloe vera* (Akinsanya et al., 2015), cacti (Fonseca-García et al., 2016), CAM plants (Citlali et al., 2018), pineapple pp (Putrie et al., 2020), and Aizoaceae (Pieterse et al., 2018). Most of these studies are exploratory and have relied only on bacterial community diversity and structure. Herein, the current study has shown the core-microbiome and culturable microbes’ function in plant growth promotion.

In the current study, we assessed both the bacterial and fungal communities of *Z. qatarensis* in the major locations where populations of this plant are naturally present. In bacterial communities, *Proteobacteria, Actinobacteria, Acidobacteria, Firmicutes*, and *Chloroflexi* were the most abundant phyla, whereas, in fungal communities, Ascomycota was the dominant phyla in the rhizosphere of *Z. qatarensis*. Recent studies on the rhizosphere microbiome of desert plants revealed a high portion of extremophilic microbes relative to stress-sensitive plants (Marasco et al., 2012). We also identified several genera of extremophilic microbes such as *Noviherbaspirillum, Marine_Group_II* and *Herpetosiphon*. Species of *Noviherbaspirillum* show considerable potential in denitrification processes in soil (Ishii et al., 2017). Sequences from the archaeal Marine group putative genus have been detected in desert environments (Pombubpa et al., 2020). Previous studies also revealed the presence of halophilic bacterial genera including *Bacillus*, *Halomonas, Halobacillus, Oceanobacillus, Marinobacter, Marinococcus*, and *Nesterenkonia* in the rhizosphere and roots of xerophytes (Etesami and Maheshwari, 2018; Mukhtar et al., 2019). These studies showed remarkably high and diverse rhizosphere colonization of *Actinobacteria, Proteobacteria, Firmicutes, Actinobacteria, Acidobacteria*, and *Bacteroidetes* (Citlali et al., 2018; Flores-Núñez et al., 2020). However, this study helps to understand the microbiome composition of wild plants growing in arid environments has not been demonstrated.

Furthermore, it is worth mentioning that soil nutrient composition was largely variable across populations from different locations, even in similar soil types. The variability was also evidenced in the salinity content and pH range alongside some of the key nutrients. Although some species of *Zygophyllum* prefer a more saline rich soil, however, we noticed that the salt contents were significantly lower in the soil of *Z*. *qatarensis*. A recent study by Wang et al. (2020) showed (*Z. brachypterum, Z. obliquum* and *Z. fabago*) resistance to 200 mM NaCl and found that the CoA biosynthesis was significantly activated in transcriptome data analysis. In addition, to salts, the Mg was significantly higher in bulk soil than in *Z*. *qatarensis* rhizosphere. This can be attributed to the overall abundance of Mg across empty quarter desert (Rub’al Khali) desert (McKay et al., 2016). In contrast, the Ca content was higher in the rhizosphere (sandy) than in bulk soil. One of the most abundant nutrients in the earth’s crust, Si is higher in the rhizosphere than in the bulk soil. In the case of plant parts, Si was more abundant in roots than in shoots. The Si content in soil increases plant resistance to different biotic and biotic stresses (Epstein, 1999; Ma and Takahashi, 2002; Ma and Yamaji, 2006), including salt and drought stress (Zhu and Gong, 2014; Rizwan et al., 2015), extreme temperature stress (Ma, 2004), nutrient deficiency (Marafon and Endres, 2013), and disease incidence (Marafon and Endres, 2013; Van Bockhaven et al., 2013). The availability, distribution, and concentration of Si have also been associated with a selection of microbial players. For example, Si presence greatly influences the microbial community structure during heavy metal contamination (Zhang et al., 2021), suggesting that extreme environments tend to influence the microbial interactions of the endemic plants, which depends on soil chemical profile.

The soil texture possibly influences the selection of key players and their variability such as *Adhaeribacter*, which was significantly more abundant in sandy than in gravel bulk soils. In contrast, *Rubrobacter* was more abundant in gravel than in sandy bulk soils. Previous studies showed that changes in soil chemistry extend selective effects on bulk soil, rhizosphere and root endosphere microbial community structure (Goss-Souza et al., 2020; Lopes et al., 2021). *Adhaeribacter* has been previously detected in desert soil and is known for its higher cellulolytic activities (Zhou et al., 2018). *Rubrobacter* – a member of the Actinobacteria phylum – was the most abundant soil microbial taxon. In the rhizosphere, many bacterial genera were enriched in sandy compared to gravel soils, mainly *Bacillus*. There is several *Bacillus* spp. —either associated with rhizosphere or endosphere showing remarkable plant growth-promoting and stress tolerance traits. The function of *Bacillus* spp. for *Z. qatarensis* has not been fully explored, which is an important question for microbiome studies in extreme environments.

Extremophilic microbes associated with desert plant species can extend their plant-growth-promoting and stress resistance traits for other plants, such as crops. Previous studies have evaluated the microbiome (especially bacterial communities) from arid soil (Jorquera et al., 2016; Citlali et al., 2018; Delgado-Baquerizo et al., 2018; Mandakovic et al., 2018; Araya et al., 2020; Astorga-Eló et al., 2020; Khan et al., 2020). However, little is known about the function of phytomicrobiome for improving crop growth and resistance to stress. Despite a few extremophile species that have been identified and characterized from desert environments (Yadav et al., 2021), their function in crop-stress tolerance has not been fully explored.

## Conclusion

The geographic pattern of *Z*. *qatarensis* microbial communities was established in this study for the first time. The composition and diversity of microbial communities varied between geographical location and soil textures. Each component had different impacts on different microbial groups, and the biogeographic distribution was the consequence of the cumulative effects of all influencing factors. Soil texture had the greatest impact on both bacterial and fungal communities, with specific microbial taxa co-occurring with sand and gravel and rhizosphere and endosphere. Furthermore, the narrow composition of core-microbiome in different compartments especially in bulk soil, shows passive selectivity of desert plants toward a large aggregation of microbial resources. This could be due to plant’s ability to utilize less energy in the form of root exudation to allow or not mutualistic relationships. These plants already focus more effort on reducing the impact of abiotic stress such as high heat, low moisture, and lack of nutrients. It has also been argued that desert plant species establish a large rhizo-sheath – a feature that may be an important adaptation to water-stressed environments (Marasco et al., 2012; Ndour et al., 2020). However, we have not seen any visible sign of rhizosheat around the roots of *Z*. *qatarensis*. The selectivity of microbial diversity and function is one of the major challenges to identify stress tolerance traits in desert plants. Hence, isolation and identification of culturable rare species can be essential to enhance functional roles in water-stressed environments, where mobilization of nutrients such as Si could essentially improve plant growth performance of not only their natural host, but several crop species. Utilizing rare players from the plant microbiome may be vital in reducing crop stress tolerance and productivity during harsh environmental conditions.

## Data Availability Statement

The original contributions presented in the study are publicly available. This data can be found here: National Center for Biotechnology Information (NCBI) BioProject database under BioProject PRJNA771947 (https://www.ncbi.nlm.nih.gov/search/all/?term=PRJNA771947) and PRJNA767523 (https://www.ncbi.nlm.nih.gov/search/all/?term=PRJNA767523) for bacteria and fungi, respectively.

## Author Contributions

AK designed the study and wrote the manuscript. SB extracted gDNA from samples. LL and AK analyzed the sequence data and prepared graphs. SA, VB, and KC cleaned the sequence data and edited the manuscript. AA-H, AA-R, and DS supervised the work and edited the manuscript. All authors contributed to the article and approved the submitted version.

## Conflict of Interest

The authors declare that the research was conducted in the absence of any commercial or financial relationships that could be construed as a potential conflict of interest.

## Publisher’s Note

All claims expressed in this article are solely those of the authors and do not necessarily represent those of their affiliated organizations, or those of the publisher, the editors and the reviewers. Any product that may be evaluated in this article, or claim that may be made by its manufacturer, is not guaranteed or endorsed by the publisher.
