# Corrigendum: Microbiome variation across populations of desert halophyte *Zygophyllum qatarensis*

**DOI:** 10.3389/fpls.2023.1156856

**Published:** 2023-02-20

**Authors:** Abdul Latif Khan, Lucas Dantas Lopes, Saqib Bilal, Sajjad Asaf, Kerri M. Crawford, Venkatesh Balan, Ahmed Al-Rawahi, Ahmed Al-Harrasi, Daniel P. Schachtman

**Affiliations:** ^1^ Department of Engineering Technology, College of Technology, University of Houston, Sugar Land, TX, United States; ^2^ Natural and Medical Sciences Research Centre, University of Nizwa, Nizwa, Oman; ^3^ Department of Agronomy and Horticulture, Centre for Plant Science Innovation, University of Nebraska-Lincoln, Lincoln, NE, United States; ^4^ Department of Biology and Biochemistry, College of Natural Science and Mathematics, University of Houston, Houston, TX, United States

**Keywords:** microbiome, desert succulents, *Zygophyllum qatarensis*, microbial communities, microbial diversity, core-microbiome

In the published article, there was an error in **Materials and Methods**, *‘DNA Extraction, Library Preparation, and MiSeq Sequencing’*, paragraph two. The accession numbers in the sentence “All quality reads related to the study are available at NCBI under BioProject PRJNA337739, 16S Accessions (KDUM00000000, KDUL00000000, and KDUK00000000), and ITS Accessions (KDUJ00000000, KDUI00000000, and KDUH00000000)” were incorrect. The sentence has been updated as follows:

“All quality reads related to the study are available at NCBI under BioProject PRJNA771947 (SRP341951) and PRJNA767523 (SRP339516) for bacteria and fungi, respectively.”

There was also an error in the **Data Availability Statement**. The accession number was incorrectly given as “PRJNA337739”. This section has been updated to read:

“The original contributions presented in the study are publicly available. This data can be found here: National Center for Biotechnology Information (NCBI) BioProject database under BioProject PRJNA771947 (https://www.ncbi.nlm.nih.gov/search/all/?term=PRJNA771947) and PRJNA767523 (https://www.ncbi.nlm.nih.gov/search/all/?term=PRJNA767523) for bacteria and fungi, respectively.”

The authors apologize for these errors and state that this does not change the scientific conclusions of the article in any way.

