# Supplementary material for: Microbiome Variation Across Populations of Desert Halophyte Zygophyllum qatarensis
Source: Front Plant Sci. 2022 Mar 31;13:841217. doi: 10.3389/fpls.2022.841217 (PMC9009292; doi:10.3389/fpls.2022.841217)
Supplement: Supplementary file 1 [file Data_Sheet_1.pdf]

**Microbiome variation across populations of desert halophyte *Zygophyllum qatarensis***

**Abdul Latif Khan[1,2#], Lucas Dantas Lopes[3#], Saqib Bilal[2], Sajjad Asaf[2], Kerri M. Crawford[4], Venkatesh Balan[1], Ahmed Al-Rawahi[2], Ahmed Al-Harrasi[2*], Daniel P. Schachtman[3*]**

[1] Department of Engineering Technology, College of Technology, University of Houston, Sugar Land, TX 77479, USA

[2]Natural and Medical Sciences Research Centre, University of Nizwa, Nizwa, 616 Oman

[3]Department of Agronomy and Horticulture and Centre for Plant Science Innovation, University of Nebraska-Lincoln, Lincoln 68588, USA

[4]Department of Biology and Biochemistry, College of Natural Science and Mathematics, University of Houston, TX 77204, USA

**Running Title**: Microbiome of *Zygophyllum qatarensis*

***Correspondence**: aharasi@unizwa.edu.om, Daniel.schachtman@unl.edu,

#Authors contributed equally to the write-up of the manuscript

**Supplementary Table 1:** Location data of different samples for Zygophyllum qatarensis Hadidi

| Population Code | Area Name | Geo Data | Soil conditions |
|---|---|---|---|
| T1P1 | Ibri | 23°24'40.3"N 56°14'54.3"E | Desert Sandy |
| T1P2 | Ibri | 23°25'35.9"N 56°16'29.4"E | Desert Sandy |
| T1P3 | Ibri | 23°25'18.9"N 55°46'38.5"E | Desert Sandy |
| T3P1 | Bidya | 22°51'57.1"N 57°46'33.1"E | Desert Sandy |
| T3P3 | Bidya | 22°51'36.1"N 57°46'30.2"E | Desert Sandy |
| T3P2 | Bidya | 22°51'23.4"N 57°45'56.1"E | Desert Sandy |
| T2P1 | Izki | 22°28'34.9"N 58°45'49.1"E | Gravel loamy |
| T2P2 | Izki | 22°28'57.9"N 58°46'34.9"E | Gravel loamy |
| T2P3 | Izki | 22°28'38.9"N 58°46'22.9"E | Gravel loamy |
| T5P1 | Themsa | 22°47'56.3"N 57°28'59.0"E | Gravel loamy |
| T5P2 | Themsa | 22°47'08.5"N 57°28'42.3"E | Gravel loamy |
| T5P3 | Themsa | 22°46'22.7"N 57°27'56.8"E | Gravel loamy |

**Supplementary Table 2:** *Z. qatarensis* and its population growth across different climatic parameters

| Parameters | T1 | T3 | T2 | T5 |
|---|---|---|---|---|
| **Temperature (ºC)** | 46 | 46 | 42 | 43 |
| **Texture** | Sandy | Sandy | Gravel | Gravel |
| **EC (dS m−1)** | 39 | 27 | 48 | 52 |
| **pH** | 7.1 | 7.2 | 6.8 | 6.7 |
| **Nitrates (mg/kg)** | 1.2 | 1.8 | 4.4 | 5.2 |

**Fig S1:** Experimental design used for this study

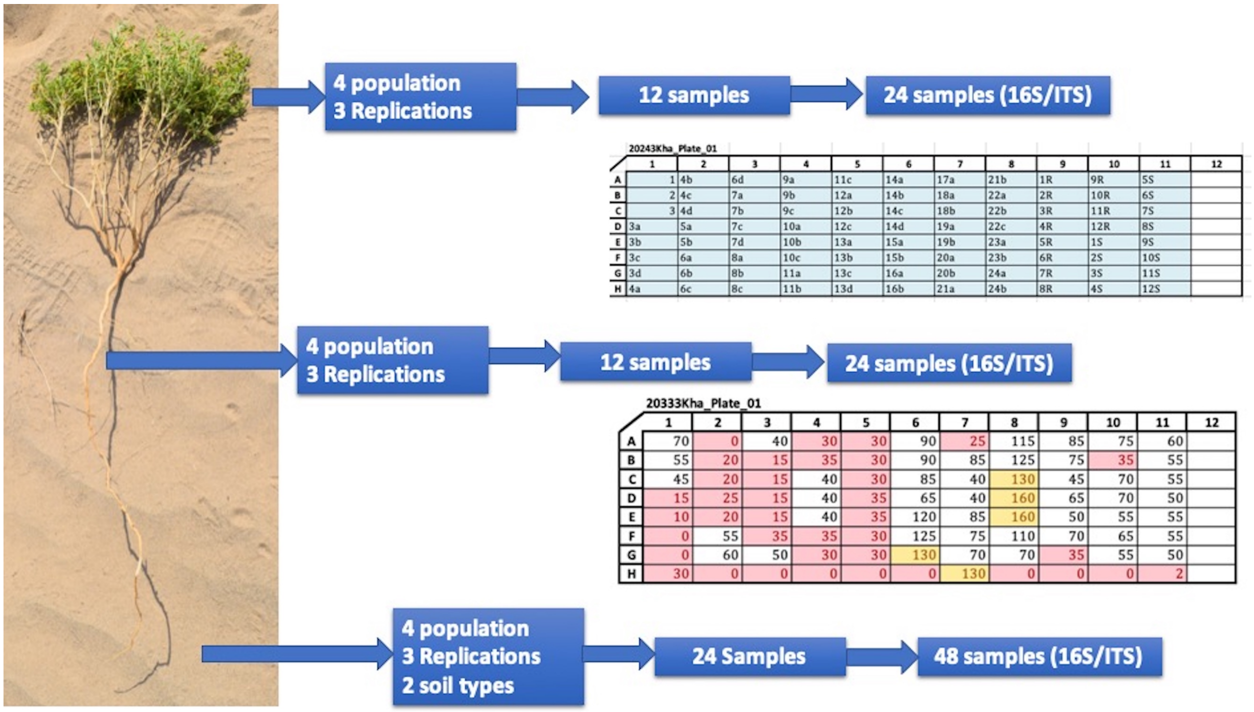

**Fig S2:** Bioanalyzer analysis of libraries of samples collected from soil, rhizosphere, and endosphere of *Z. qatarensis*

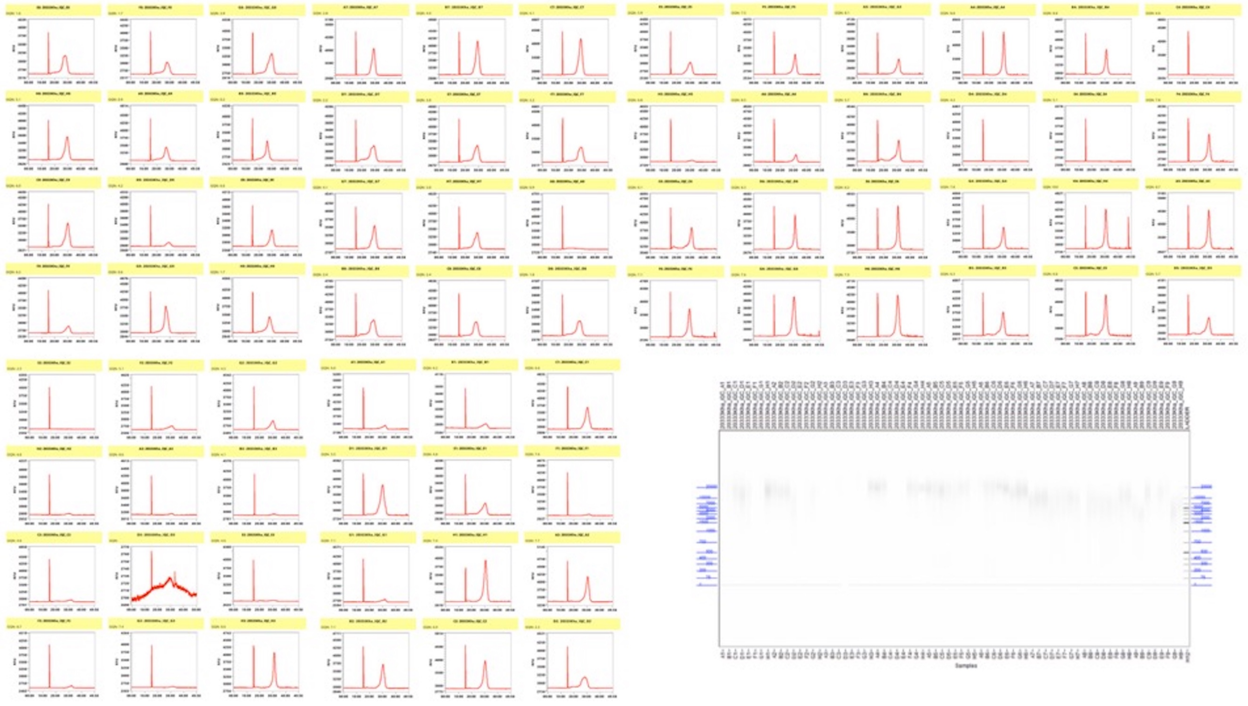

**Figure S3:** Distribution of microbial counts across rhizosphere and endospheric regions

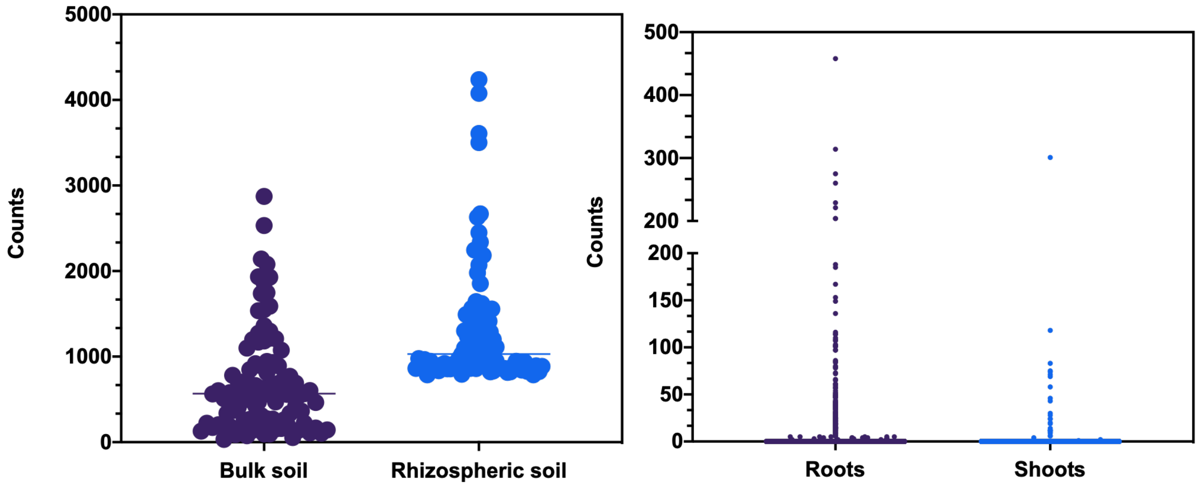

**Fig S4:** Bacterial species distribution across different samples and taxonomical classification.

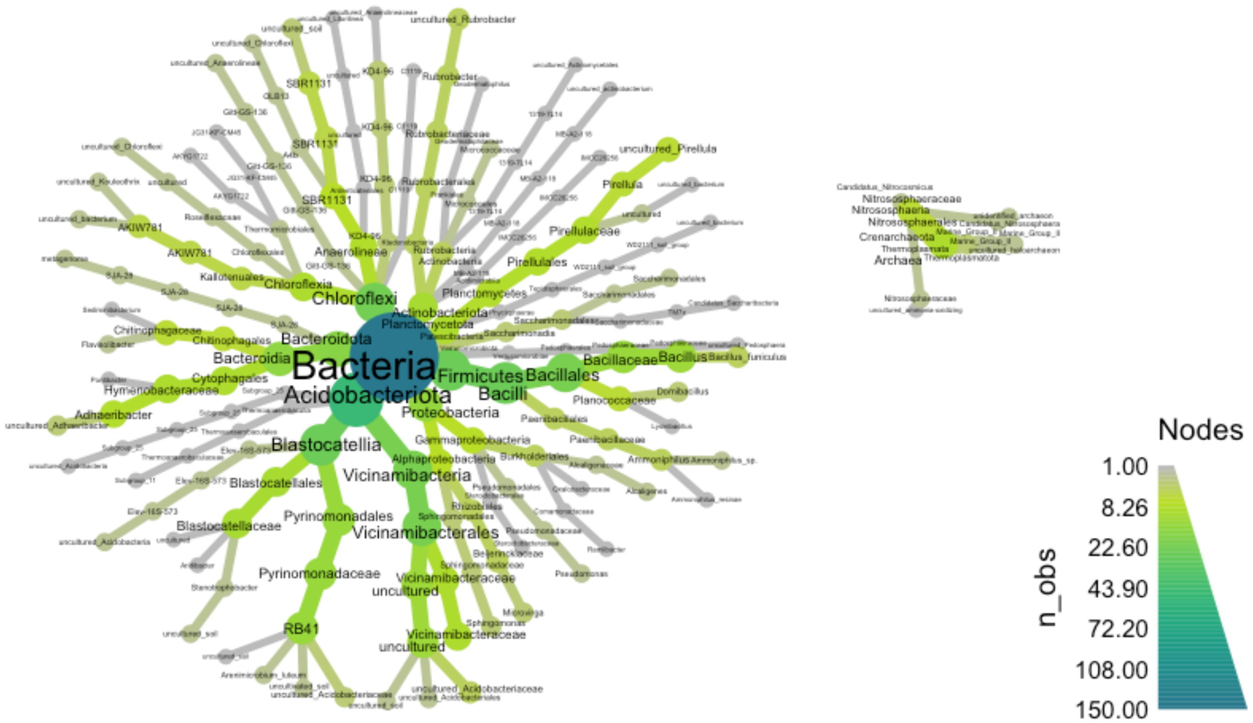


**Fig S5** Heat map showing the abundances and distribution of bacterial communities across representative soil types

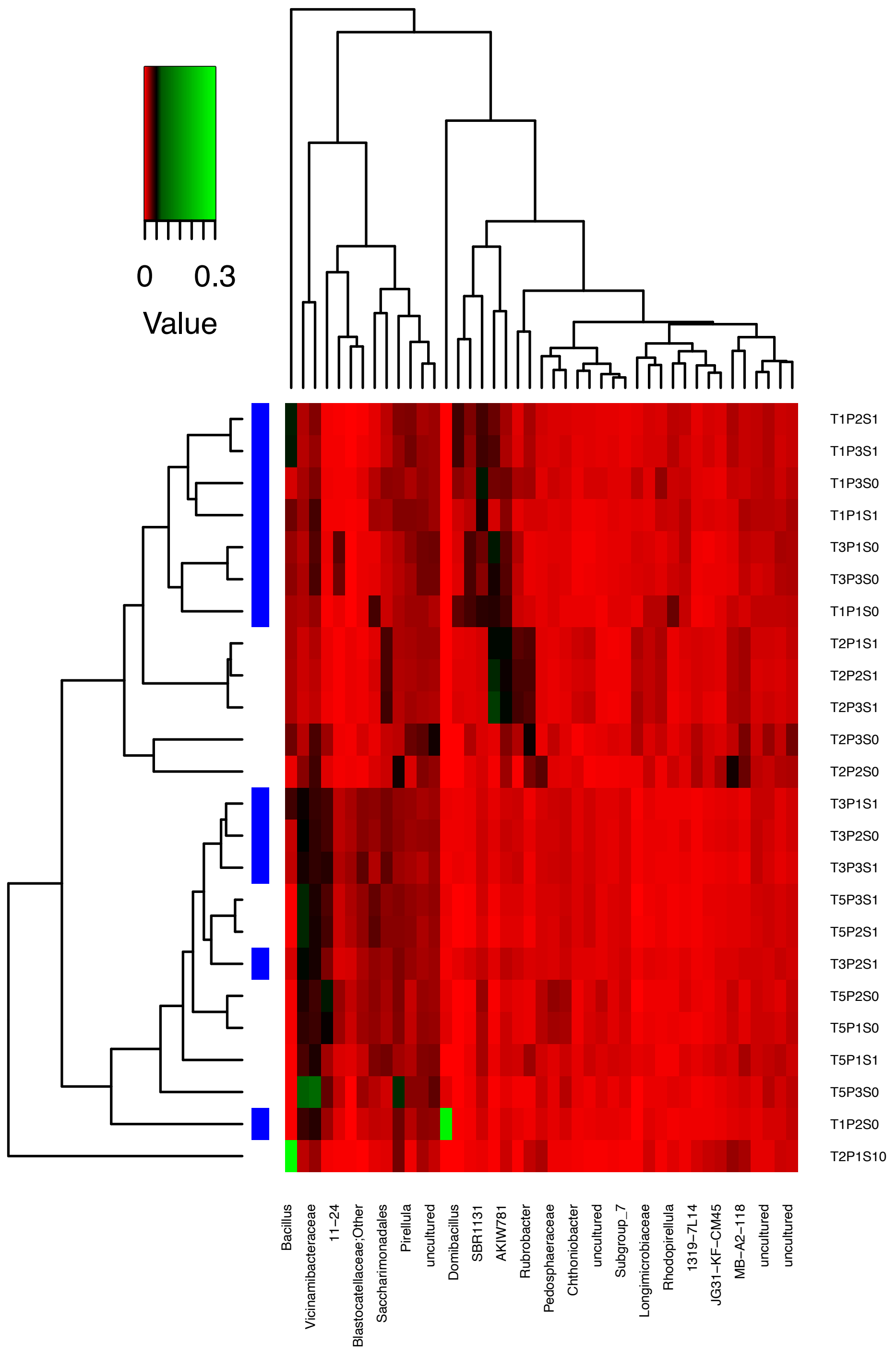
0
0.3
Value
T1P2S1
T1P3S1
T1P3S0
T1P1S1
T3P1S0
T3P3S0
T1P1S0
T2P1S1
T2P2S1
T2P3S1
T2P3S0
T2P2S0
T3P1S1
T3P2S0
T3P3S1
T5P3S1
T5P2S1
T3P2S1
T5P2S0
T5P1S0
T5P1S1
T5P3S0
T1P2S0
T2P1S10
Bacillus
Vicinamibacteraceae
11-24
Blastocatellaceae;Other
Saccharimonadales
Pirellula
uncultured
Domibacillus
SBR1131
AKIW781
Rubrobacter
Pedosphaeraceae
Chthoniobacter
uncultured
Subgroup_7
Longimicrobiaceae
Rhodopirellula
1319-7L14
JG31-KF-CM45
MB-A2-118
uncultured
uncultured

**Fig S6:** Fungal distribution across soil and endosphere.

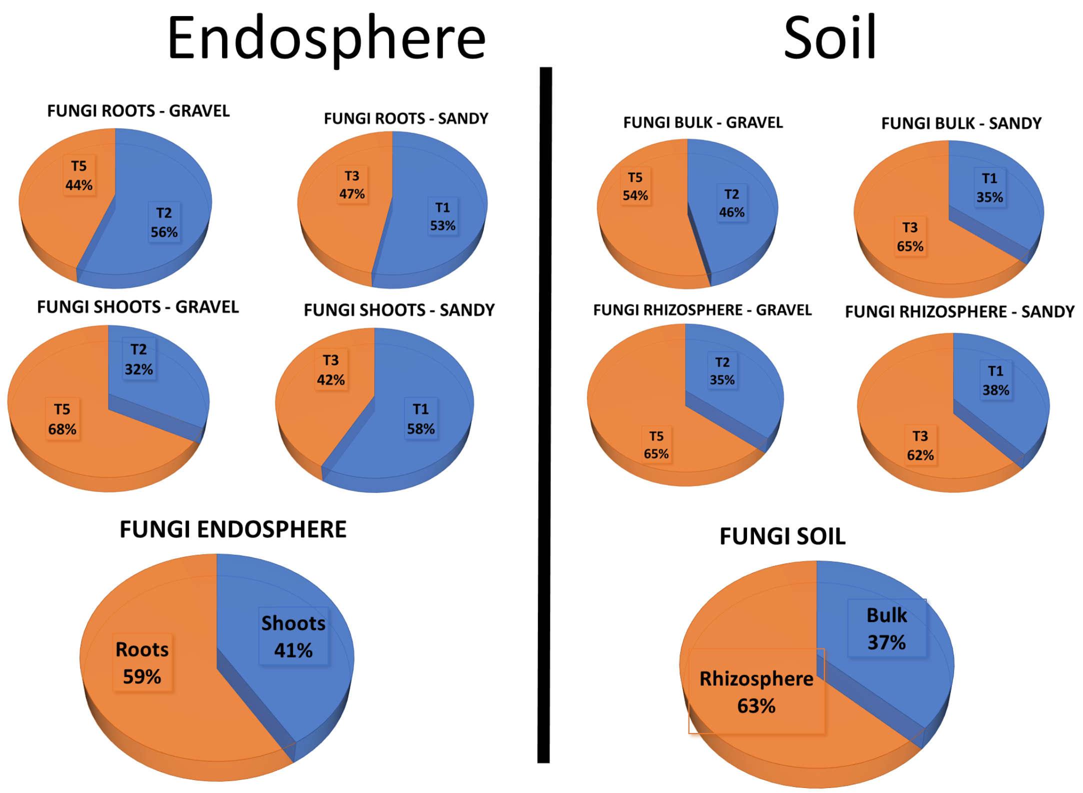


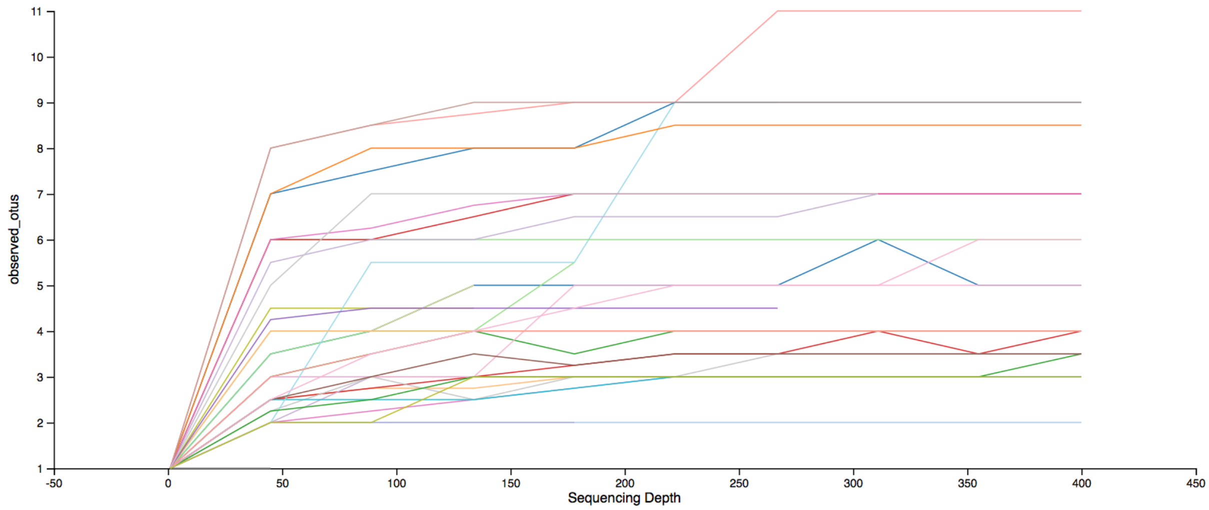

Figure S7: The Rarefaction curve of microbial communities of different samples and sequencing depth to reflect the fungal diversity.

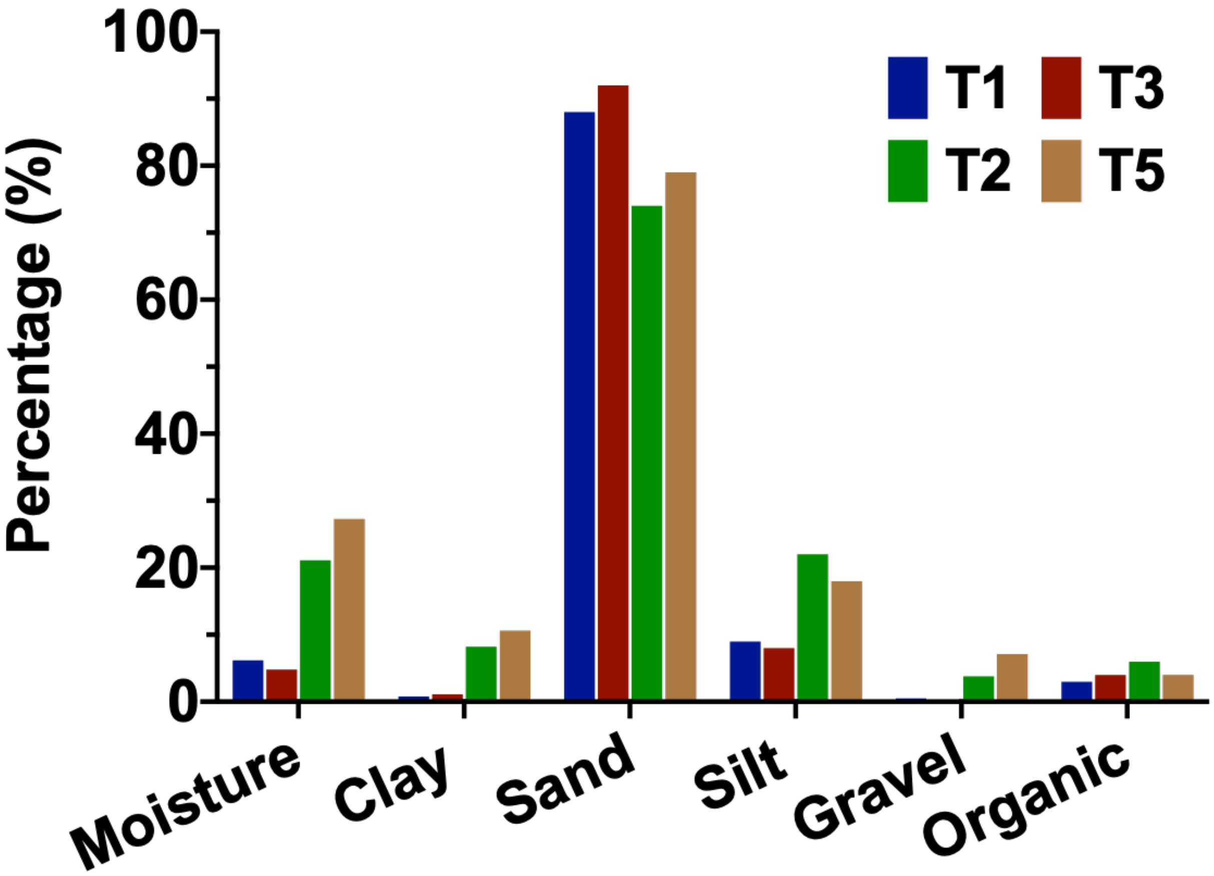


| Source of Variation | % of total variation | P value | P value summary | Significant? |
|---|---|---|---|---|
| Population | 95.89 | <0.0001 | **** | Yes |
| Soil | 0.8928 | 0.2844 | ns | No |

Figure S8: Soil physical and morphological properties of different populations of *Z. qatarensis*
